# Molecular Mechanisms Provide a Landscape for Biomarker Selection for Schizophrenia and Schizoaffective Psychosis

**DOI:** 10.3390/ijms242015296

**Published:** 2023-10-18

**Authors:** Stephanie Fryar-Williams, Jörg Strobel, Peter Clements

**Affiliations:** 1 Youth in Mind Research Institute, Unley Annexe, Mary Street, Unley, SA 5061, Australia; 2 Department of Nanoscale BioPhotonics, School of Biomedicine, Faculty of Health and Medical Sciences, The University of Adelaide, Adelaide, SA 5000, Australia; 3 Department of Psychiatry, Adelaide Medical School, Faculty of Health and Medical Sciences, The University of Adelaide, Adelaide, SA 5000, Australia; jorg.strobel@adelaide.edu.au; 4 Department of Paediatrics, Adelaide Medical School, Faculty of Health and Medical Sciences, The University of Adelaide, Adelaide, SA 5000, Australia; peter.clements@adelaide.edu.au

**Keywords:** biomarkers, psychosis, schizophrenia, bipolar, epigenetics, methylation, vitamin B6, riboflavin, catecholamines, *MTHFR C677T* genotype

## Abstract

Research evaluating the role of the 5,10-methylenetetrahydrofolate reductase (*MTHFR C677T*) gene in schizophrenia has not yet provided an extended understanding of the proximal pathways contributing to the 5-10-methylenetetrahydrofolate reductase (MTHFR) enzyme’s activity and the distal pathways being affected by its activity. This review investigates these pathways, describing mechanisms relevant to riboflavin availability, trace mineral interactions, and the 5-methyltetrahydrofolate (5-MTHF) product of the MTHFR enzyme. These factors remotely influence vitamin cofactor activation, histamine metabolism, catecholamine metabolism, serotonin metabolism, the oxidative stress response, DNA methylation, and nicotinamide synthesis. These biochemical components form a broad interactive landscape from which candidate markers can be drawn for research inquiry into schizophrenia and other forms of mental illness. Candidate markers drawn from this functional biochemical background have been found to have biomarker status with greater than 90% specificity and sensitivity for achieving diagnostic certainty in schizophrenia and schizoaffective psychosis. This has implications for achieving targeted treatments for serious mental illness.

## 1. Introduction

Schizophrenia arises from a complex range of different genetic traits, adverse developmental risk factors, and environmental effects. These accumulate and interact to produce a variety of distressing symptoms, with general onset in adolescence and youth [1].

In the past, there has been recognition that schizophrenia is linked to vitamin deficiency [2,3,4], however, the mechanisms involved are not understood in an integrated manner. Similarly, it is well known that schizophrenia is linked to neurotransmitter deficits and there has been increasing acknowledgement of the role of DNA methylation changes in epigenetic processes and schizophrenia [5,6]. Whilst this knowledge is useful, there is now a need to describe the exact linkages between vitamin deficiencies, methylation, neurotransmitter level fluctuations, and epigenetic processes. The purpose of this review is to provide a bridge of understanding between these molecular processes, and to demonstrate the broad biochemistry landscape from which biomarkers may be selected to predict diagnoses and outcomes in schizophrenia and schizoaffective disorder. 

**Scheme 1:**
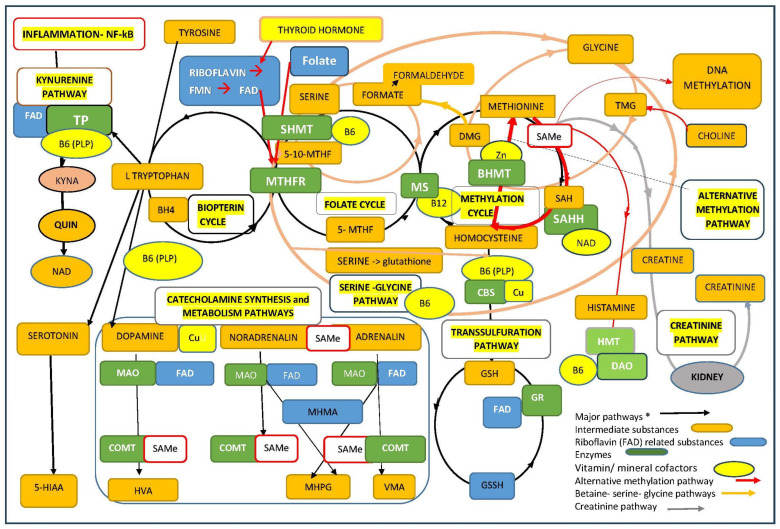

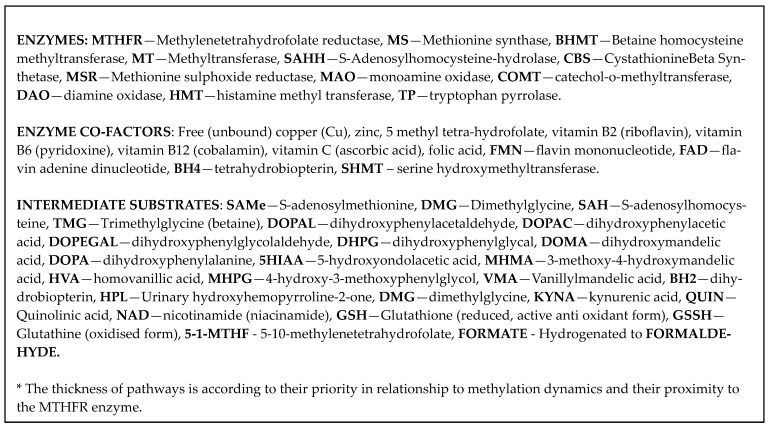
Extended biochemistry.

The *MTHFR C677T* gene polymorphism has a long-recognized relationship with schizophrenia [7,8]. However, its role in schizophrenia has been considered controversial due to failure of replication in some studies [9,10,11]. Such contradictory findings have occurred in settings where research data have not been differentiated at the genotype level. Nevertheless, it seems that more recent research is validating the role of this gene in schizophrenia [12,13,14], and this review provides an in-depth understanding of relationships in proximal and distal biochemical pathways related to MTHFR enzyme function. The integrated understanding presented is derived from repeated comprehensive searches conducted annually in five databases (PubMed, Web of Science, Scopus, Google Scholar, and Consensus AI application), between November 2015 and August 2023. Search terms used were “*MTHFR C677T* gene polymorphism”, “*MTHFR C677T* genotype”, “riboflavin (vitamin B2)”, “flavin adenine dinucleotide (FAD)”, “flavin mono nucleotide (FMN)”, “vitamin B6”, “(GSH)”, and all the intermediary substances and cofactors described in the biochemical pathways outlined in Scheme 1. This literature review resulted in the conceptualization of the integrated landscape, from which candidate markers for schizophrenia and schizoaffective disorder can be selected for biomarker validation.

The need for diagnostic biomarkers of schizophrenia and schizoaffective disorder arises because there is no typical early symptom or sign that is characteristic of psychosis onset or future development of schizophrenia or schizoaffective disorder. Unstable early symptoms often make diagnostic confirmation difficult [15,16,17,18], and such uncertainty is stressful for the patient and health care providers alike. Diagnostic dilemma may lead to a delay in diagnosis with progressive illness and poorer outcomes, including impoverished social relations and impaired family function. In this context, biomarkers that identify aberrations in the underlying biochemistry pathways are a much-required advance in psychiatry. This is because of their potential to provide objective support for symptom-based clinical assessments, inform clinical management decisions, and reduce illness progression towards psychosis [19].

## 2. Background Biochemistry

The homozygous (TT) *MTHFR 677* genotype is the most common autosomal recessive form of inherited folate metabolism disorder. It is inherited as an autosomal recessive genotype and has a reported relationship with schizophrenia [7]. The prevalence of the homozygous (TT) *MTHFR 677* genotype in global populations varies widely (2.5–34%) according to geographic latitude [20] and folic acid contents in food. It has a very high prevalence among Mediterranean countries [21,22], but a low prevalence (range 5–12%) in the white Australian population [23]. This gene relates to an array of conditions including fetal neural tube defects, preterm birth, autism, gait disturbances, cardiac disease, and colorectal cancer [24,25]. Despite this gene’s strong association with cognitive disturbance [26] and neurological diseases [27], the association of the different *MTHFR C677T* genotypes to psychosis symptoms and schizophrenia diagnosis has not been extensively investigated, and a broad basis for phenotypes has yet to emerge.

The MTHFR rate-limiting enzyme is coded for by different gene variants or genotypes of the *MTHFR C67*7T gene [17,18]. These genotypes consist of C (Cytosine) or T (Thymidine) combining at the 677 position in three possible allelic forms [24]:

- When Cytosine is replaced by Thymidine at the 677th position of the *MTHFR* gene, a genotype possessing two TT alleles is formed. This homozygous (TT) *MTHFR 677* genotype codes for an MTHFR enzyme that is easily denatured by heat. Such denaturation restricts the enzymes production of 5-methyl tetrahydrofolate (5-MTHF) for conversion of homocysteine into methionine in neuron and glia brain cells [28,29];
- When both Cytosine and Thymidine are present at position 677, the heterozygous *MTHFR 677 CT* genotype codes an enzyme with less restricted catalysis capacity; and
- When two cytosine molecules occupy this position, the homozygous *MTHFR 677 CC* genotype codes for an enzyme with unrestricted capacity to produce 5-MTHF.

### 2.1. The Importance of Riboflavin in MTHFR C677T-Related Biochemical Pathways

The modulating role of riboflavin (vitamin B2) in the methylation flux scenario cannot be underestimated because it is precursor for flavin mononucleotide (FMN) and flavin adenine dinucleotide (FAD) synthesis [30]. Riboflavin is not synthesized in human tissue but is derived directly from dietary intake and is also synthesized in the human gastrointestinal microbiome by organisms such *as lactobacilli*, *bacillus subtilis*, *E. coli*, and *saccharomyces cerevisiae* [31,32]. The essential nature of microbiome riboflavin synthesis is highlighted by the presence of highly redundant, conserved riboflavin absorption mechanisms in the gastrointestinal tract and kidney. In a setting of labile methylation, the kidney has a mechanism for modulating flavin levels through flavin reabsorption and excretion. This occurs through an adaptive bi-directional transport mechanism that can conserve flavin when it is in short supply but excrete it when in excess [33]. If these redundant riboflavin absorption and conservation mechanisms nevertheless render riboflavin in short supply, there will be insufficient precursors for FAD synthesis and insufficient FAD to act as a cofactor for MTHFR enzyme activity. Thus, there may be inadequate 5-MTHF production for fueling methylation. Similarly, there may be insufficient FMN to bring about vitamin B12 re-activation, allowing a state of MS enzyme stasis to occur [34]. Consequently, methionine reconstitution from homocysteine via MS is retarded, and supply of the methylation cycle’s main product—S adenosyl methionine (SAMe)—is reduced (Scheme 1). When 5-MTHF and SAMe supply are reduced, a low methylation state sometimes described as “under methylation” ensues. Low-SAMe-associated under methylation adversely affects the activity of over forty methyl-transferase reactions that require a supply of this methyl donor cofactor [35,36,37].

Conversely, when riboflavin procurement, absorption, and conservation processes ensure that there is a sufficient supply of riboflavin, then FAD and FMN and MTHFR and MS activity are all, respectively, facilitated. The MS enzyme can then allow 5-MTHF to donate its carbon to homocysteine to form methionine, which in turn generates S-adenosyl methionine (SAMe). As a major methylation factor in cells, SAMe facilitates RNA and DNA transcription and contributes to gene expression silencing [38]. SAMe also contributes methylation to modify RNA histones which contribute to the epigenetic regulation of gene expression [39,40]. Since the 5-MTHF molecule provided by the MTHFR enzyme is an important donor of methyl groups for SAMe synthesis from methionine via homocysteine metabolism, the *MTHFR C677 T* gene, which codes for this enzyme, makes an important contribution to the epigenetic process. Notably, SAMe is also required for histamine metabolism by the enzyme histamine methyl transferase (HMT) [41]. It is also required for catecholamine metabolism by the enzyme catechol-o-methyl transferase (COMT) [42], and for the conversion of noradrenaline (NA) into adrenaline (AD) [43], and for creatine formation from glycine [44].

### 2.2. Homozygous MTHFR 677 TT Genotype and Potential for Compensatory Mechanisms to Salvage SAMe Production by the Dynamic Mechanism of “over Methylation”

Carriers of the TT genotype suffer from intrinsic low-methylation (under-methylation) effects because of insufficient 5-MTHF for assisting downstream methionine synthase (MS). As explained above, adequate activity of MS is required to reconstitute methionine from homocysteine and thus manufacture sufficient S–adenosyl methionine (SAMe) to cofactor a wide range of methyl-accepting reactions throughout the cellular system [45]. Because SAMe is a cofactor for so many critical biochemical reactions, such as sustaining DNA methylation and maintaining multiple genes silencing [37,39], the body’s biochemistry must compensate for a *MTHFR 677 TT*-related methylation defect. A solution is achieved by activating the zinc and vitamin B6 co-factored enzyme betaine homocysteine methyl transferase (BHMT) [46,47,48,49,50]. This enzyme makes up to 1.5% of all the soluble protein of the liver and occupies a central point in the methylation cycle (Scheme 1). Therefore, in the context of insufficient riboflavin or its precursors or of *MTHFR 677 TT* inadequacy for supplying sufficient 5-MTHF methyl groups, the MS enzyme is stalled and cannot adequately convert homocysteine into methionine. In this setting, a compensative pathway for methionine synthesis, which we have called the “SAMe salvage pathway”, is activated across the methylation cycle to serve as an alternative route for homocysteine’s thiol group to be methylated. In this manner, BHMT compensates for stalled MS activity by reconstituting methionine for SAMe production. Recent evidence suggests that this alternative methylation pathway can greatly influence methionine and homocysteine homeostasis [51,52]. By salvaging methionine for SAMe production, SAMe can then act as a cofactor for COMT metabolism of catecholamines. However, if this process becomes over-driven by BHMT, there is a theoretical potential for excessive SAMe production (over-methylation), to drive COMT metabolism, to a point where catecholamine reserves are depleted. If this should occur, there is an attendant risk of reduced neurotransmission, with severe depression and suicide [53].

### 2.3. The Mechanism of the Zinc and Copper Trace Element Relationship with MTHFR 677 TT Genotype

Since zinc is a cofactor for the BHMT enzyme, excessive compensative use of the previously mentioned SAMe salvage pathway may result in zinc depletion over time. Then, as a reciprocal homeostatic relationship exists between low zinc and high free copper [54,55], compensatively elevated free copper exerts an inhibitory effect on the activity of the cystathionine beta synthase (CBS) enzyme [56]. CBS lies beneath homocysteine in the top section of the of the trans-sulfuration (TSF) pathway (Scheme 1), which culminates in the synthesis of the powerful antioxidant glutathione [57]. In this inhibitory setting, homocysteine may become trapped and elevated, with its elevation exacerbated if homocysteine’s major metabolic pathway (through MS to methionine) is also compromised by insufficient 5-MTHF MS co-product [58]. If compensative activation of the BHMT pathway then arises, this sets up a vicious cycle whereby homocysteine metabolism is inhibited from several directions at once. These mechanisms explain the well-documented research findings of homocysteine level elevation in the presence of the *MTHFR 677* homozygous genotype [59,60]. Homocysteine elevation may be further exacerbated when its other metabolizing enzyme (CBS) is inhibited in the presence of elevated free copper levels. This is likely to occur in the afore-mentioned compensative BHMT pathway, because this enzyme utilizes zinc as a cofactor. If zinc is over-utilized in this manner, it can leverage high copper ion levels due to an indirect reciprocal relationship between zinc and copper through their metallothionein-binding effects [54,55]. Elevated free copper can in turn cofactor dopamine beta decarboxylase to convert dopamine (DA) into noradrenaline (NA) [61]. Additional information is provided in the literature regarding the cofactor role of copper in the conversion of dopamine into noradrenaline and its contrasting inhibitory role for the CBS enzyme in the trans-sulfuration pathway to glutathione synthesis [62,63].

### 2.4. The Potential for the MTHFR 677 TT Genotype to Increase Bioavailability of Flavin and Vitamin Molecules

An unexpected beneficial side-effect of the homozygous *MTHFR 677 TT* genotype is that the flavin adenine nucleotide (FAD) cofactor is displaced from the thermolabile, low -activity enzyme that is coded by this genotype [64]. Therefore, together with its flavin adenine dinucleotide (FMN) precursor, FAD is more available to act as a critical cofactor for many other enzymes. FAD and FMN are derived from riboflavin (vitamin B2). After gastrointestinal absorption, riboflavin is converted into FMN by the enzyme riboflavin flavokinase, which attaches a phosphate group on the alpha-carbon of riboflavin’s ribityl side chain. In humans, riboflavin flavokinase is primed by thyroid hormones [65] and is a magnesium dependent enzyme. The next enzyme in the chain is FAD synthase, which attaches an ADP (adenosine diphosphate) to FMN’s ribose moiety to synthesize FAD. This serves as a cofactor for the MTHFR enzyme [66,67].

### 2.5. Dynamic Mechanisms between Riboflavin Derivatives, FMN, and FAD and Vitamin B6

Several biochemical vitamin activation pathways are routinely facilitated by the riboflavin derivatives FMN and FAD. For instance, FMN activates vitamin B12 [34,68,69,70] and assists in the conversion of vitamin B6 into its biologically active pyridoxine-5-phosphate (PLP) cofactor form [68]. This allows PLP to cofactor synthesis of serotonin from L tryptophan [71] and to also participate in the B6-dependent trans-sulfuration (TSF) pathway [72]. In this pathway, PLP also takes the opportunity to activate cystathionine beta synthetase (CBS), the enzyme that metabolizes homocysteine and thereby contributes to reduced glutathione production via the powerful trans-sulfuration pathway. Reduced glutathione (GSH) is a major redox agent for cells [57]. Coincidentally, if there is insufficient activation of vitamin B6 by FAD, serine’s conversion into glycine is inhibited [73]. In this setting, serine is conserved and its entry into the upper trans-sulfuration pathway can provide a back-up mechanism for maintaining glutathione formation. Thus, the joint contribution of homocysteine and serine at the upper portion of the trans-sulfuration pathway steers around the problem of any insufficiently activated vitamin B6 cofactor impairing the activity of enzymes that influence homocysteine’s progress down this pathway.

Apart from these interesting remote influences, PLP also participates in many other notable enzyme reactions. It cofactors the conversion of homocysteine into methionine via BHMT [74], assists synthesis of dopamine (DA) from L dopa [75,76], influences the levels of both serine and tryptophan [35,77,78], and metabolizes L tryptophan through tryptophan pyrrolase activation [79].

Meanwhile, FAD cofactors are facilitating the monoamine oxidase (MAO) enzyme which catalyzes the first step of catecholamine metabolism [80]. If FAD is unavailable, catecholamines are poorly metabolized, meaning that NA and AD levels are higher than that of their common metabolite MHMA. In this context, the ratio of NA/MHMA and AD/MHMA is likely to be high [81]. Moreover, since FAD is unutilized by the inactive MTHFR enzyme [30], it is readily available to cofactor the activity of MAO. This enzyme undertakes the first step of catecholamine degradation [82]. Then, the second step of catecholamine degradation is undertaken by the catechol-o-methyltransferase (COMT) enzyme which requires SAMe as a cofactor [83]. In this context, even though MAO activity is FAD-facilitated, SAMe supply is not plentiful, allowing neurotoxic catecholamine intermediate semi-quinone substances to accumulate and damage neurons [84]. This is a further reason why methionine needs to be reconstituted from homocysteine using the BMHT pathway and utilizing trimethyl glycine (betaine) as the methyl donor molecule [46,47,48,49,50]. By salvaging methionine for SAMe production, SAMe can then act as a cofactor for COMT metabolism of catecholamines. Of note, however, is the potential for excessively driven catecholamine metabolism to deplete catecholamine reserves, with attendant risk of severe depression and suicide [53].

FAD also plays an important role in the reconversion of oxidized glutathione (GSSH) back to its active, reduced form (GSH) [85]. Maintenance of this reduced form of glutathione is imperative to dampen excessive free oxygen radical formation, that is, it is a key component of schizophrenia pathology [86]. A further role for FAD occurs in facilitating the monoamine oxidase enzyme involved in serotonin’s metabolism into 5 hydroxy indole acetic acid (5HIAA). Such metabolism may be boosted in carriers of the MTHFR 677 TT genotype, where FAD is unutilized by the inactive MTHFR enzyme [80].

### 2.6. Dynamic Mechanisms between Flavin Molecule, Vitamin B6 and Nicotinamide 

At the top end of the kynurenic pathway, vitamin B6 (PLP) and the reduced form of flavin mononucleotide (FMNH2) jointly influence tryptophan metabolism by activating the enzyme tryptophan pyrrolase (also known as tryptophan 2,3-dioxygenase (TDO) or indoleamine 2,3-dioxygenase (IDO)) [79]. A chain of reactions then leads down this pathway to nicotinamide (NAD/NADP/vitamin B3) synthesis [87,88] (Scheme 1). In an interesting remote mechanism, riboflavin and FMN levels are indirectly modulated through NADH, which competes for binding sites on the pyrophosphatase enzyme. This enzyme plays a role in inhibiting FAD metabolism with consequent conservation of its flavin precursors [89]. This dynamic is more likely to occur when serine is held back from conversion into 5-MTHF by the reduced activity of the *MTHFR 677 TT* -coded MTHFR enzyme. This allows serine’s vitamin B6-dependent metabolism to glycine to be boosted along with glycolysis [90]. Glycolysis can in turn boost the polyol pathway [91] and, through that remote mechanism, enhance a feedback pathway to riboflavin maintenance by supporting its synthesis from guanosine triphosphate (GTP) along this pathway [92]. NAD can also be phosphorylated into NADP and then reduced into various NADPH molecules. NADP and FAD then collaborate to facilitate the ferredoxin reductase enzyme, thereby playing a role in iron transport mechanisms and heme formation [93,94]. NADP assists metabolism of heme-containing cytochrome P450 cytochromes [95] and plays a final role in heme degradation by the biliverdin reductase enzyme [96]. Furthermore, reduced forms of NAD and FAD (NADH and FADH2) donate their electrons to ATP generation in the electron transport chain [97] and as a further contribution to body chemistry, NADPH assists the vitamin D activation process [98,99].

Conversely, it is notable that after vitamin B6 and FAD facilitate the kynurenine pathway to nicotinamide (NAD) synthesis [79,87,88], NAD plays a key role in the methylation cycle by co-factoring the enzyme SAH hydrolase (SAHH). This enzyme has a reaction equilibrium that favors SAH production over homocysteine (HCY) production [100]. However, SAH is a methylation suppressor, due to its action as a feedback inhibitor of SAMe synthesis from methionine [101]. Moreover, SAH is a competitive inhibitor of creatine biosynthesis from glycine [102]. By both these means, SAH trapping, and accumulation brings about a decrease in creatine and creatinine production [103], (Scheme 1). Furthermore, an intermediate substrate in the formation of creatine, (guanidinoacetate), also exerts feedback inhibition on SAMe formation with methylation suppression effects that extend as far back as homocysteine and BHMT. Since 70% of SAMe-derived methyl groups are required for the methylation of guanidinoacetate, excessive methylation-driven creatinine levels can act to further inhibit SAMe production, providing a feedback loop with potential to exacerbate any pre-existent undermethylation state [104].

## 3. Discussion

This review of biochemical theory as it relates to riboflavin, methylation, and upstream and downstream regulated pathways, is derived from a review of current research and professional opinions about the molecules contained in these pathways and their proximally interactive mechanisms. When these proximal influences are integrated with more distal influences, they form a wider landscape for selecting candidate biomarkers. This allows a functional understanding of the relationship between vitamin deficiencies, neurotransmitter fluctuations, and epigenetic processes. Wherever possible, we have reviewed the data for consensus about inter-relationships and wider issues related to methylation dynamics; however, most reported studies have not been replicated, imposing a caveat that the explanatory dynamics described in this review can only be taken as a guideline. For instance, there is still insufficient pre-clinical and clinical data on *MTHFR C677T* gene functions in relationship to other psychiatric disorders. Moreover, since schizophrenia is a condition influenced by multiple genes and genotypes [105,106], it cannot be assumed that the *MTHFR C67TT* gene is the only gene influencing trajectories and outcomes in schizophrenia and psychotic disorders. For all these reasons, the opinions given in this review may be subject to change with the accumulation of more data.

This review has not addressed specific biomarker roles within the other CT and CC genotypes in detail, as these are the subject of other publications, for which this review of the wider methylation-related biomarker landscape may provide helpful, supportive information. Neither has this review addressed the role of glutamate in schizophrenia or its effects upon both N methyl D acetylase (NMDA) receptor function and AMPA receptor function, for which there is now a wide body of relevant knowledge [107,108,109,110,111,112]. Since the tricarboxylic acid (TCA) cycle is fed by pyruvate from glycolysis, in a pathway that requires activated vitamin B6, lower vitamin B6 activation by unavailable riboflavin (vitamin B2), could be expected to inhibit downstream glutamate formation from alpha ketoglutarate, as an offset of the TCA cycle [113,114]. Thus, low vitamin B6 activation, occurring in a setting of low riboflavin [67] and low methylation [81], may be one explanation for previously reported research findings of low glutamate, and low global NMDA receptor function, in schizophrenia [115,116]. A further NMDA receptor activator in schizophrenia is D-serine. D-serine is formed from L-serine by the enzyme serine racemase (SR), which is a vitamin B6 (PLP)-dependent enzyme. This leads to the hypothesis that in low methylation states, a D-serine deficit may contribute to low glutamate and the reported NMDA hypoactivation in schizophrenia [117,118].

Scheme 1 displays pathways related to serine and glycine, which include trimethyl glycine (TMG) and Dimethyl glycine (DMG). Serine is a remarkable regulator molecule which is worthy of biomarker status because it not only produces precursor molecules for MTHFR conversion into 5-MTHF, but also plays a background role in multiple other molecular pathways. With the assistance of vitamin B6 cofactor, serine is metabolized and converted into glycine [119]. Serine can also be decarboxylated to form ethanolamine, which is a precursor for choline synthesis [120]. Choline is then a precursor for trimethyl glycine (TMG) synthesis, with TMG then demethylated by the BHMT enzyme to form dimethyl glycine (DMG). After this, DMG is recycled back to glycine. DMG is also an interesting molecule with the potential to activate the NMDA receptor via its glycine site [121].

A further function of serine is its participation in the trans-sulfuration pathway (Scheme 1), where it serves as an alternative substrate to homocysteine for CBS manufacture of downstream glutathione (GSH). This serine function is important when homocysteine synthesis is held up in low-methylation states. Though it is not possible to include all serine metabolic pathways in Scheme 1, together with its activated vitamin B6 cofactor, serine participates in several other important pathways. For instance, it contributes to glycolysis [122] and synthesis of phosphatidyl serine for cell membrane maintenance [123]. Within cell mitochondria, serine’s end-product glycine condenses with the citric acid cycle molecule succinyl CoA. This forms 5′-Aminolevulinic acid (ALA) via the vitamin B6 (PLP)-dependent 5′-Aminolevulinic acid synthase (ALA-S) enzyme that regulates heme synthesis in the liver and erythroid cells. ALA then exits the mitochondria and within cell cytosol, condenses two of its rings to form the pyrrole ring of porphyrin, using porphobilinogen synthase, which utilizes zinc as a cofactor [124]. From there, there are a succession of steps to form haeme porphyrin [125]. Haeme porphyrin is then degraded by haeme oxidase in an oxidative process that is conjectured to contribute to urine excretion of the porphyrin pyrrole biomarker, hydroxypyrroline-2-one (HPL). High levels of this molecule have been detected in the urine of patients with schizophrenia and particularly in those who do not possess the homozygous *MTHFR 677* polymorphism [81,126]. Elevated urine HPL levels are reportedly reduced by vitamin B6 (PLP) and zinc supplementation, though the exact mechanism of this effect is yet to be established [127]. Given the relevance of many of the above-discussed downstream effects of the serine–glycine pathway for schizophrenia, serine and glycine and HPL have potential roles as biomarkers for schizophrenia, and preliminary research on their role in this disorder has indeed been reported in the literature [128].

Also implicated in schizophrenia is the wide panoply of inflammatory and immune response factors [129,130]. These factors include the effects of nuclear transcription factor -kappaB ‘s (NF-κB) activation of tryptophan pyrrolase (indolamine 2,3-dioxygenase: IDO) enzyme at the top of the kynurenine pathway (Scheme 1) and the gastrointestinal inflammatory effects of histamine and formaldehyde. The inflammatory effect of vitamin B2 deprivation on the gastrointestinal tract is a further interesting consideration.

As briefly discussed in Section 2.5 of this review, the vitamin B6 (PLP), FAD, and the haem-dependent enzyme tryptophan pyrrolase (indolamine 2,3-dioxygenase: IDO) metabolize L tryptophan, which is serotonin’s precursor substrate (Scheme 1). The inflammatory role of NF-κB is well studied, and molecular alterations in this kynurenine pathway have been related to schizophrenia [131]. Through NF-κB activation of tryptophan pyrrolase [132], tryptophan metabolism is enhanced, effectively carrying out a tryptophan steal operation that makes this molecule less available as a precursor for serotonin synthesis [133,134,135]. The tryptophan pyrrolase enzyme is vitamin B6 (PLP)- and FAD-dependent [79]. Since it can be expected that gastrointestinal transport and absorption of vitamin B6 and the riboflavin FAD precursor will be inhibited in inflammatory conditions, FAD’s considerable influence in promoting two enzymes in the kynurenine pathway may be diminished [136,137]. If kynurenine pathway activity is restricted in this manner, unmetabolized L tryptophan may be more available to act as a precursor for serotonin synthesis. These dynamics may explain the related findings of low riboflavin and elevated 5-HIAA excretion in carriers of the *MTHFR 677 CC* genotype [81]. In this context, it is noted that increased plasma levels of tryptophan, serotonin, and 5-HIAA in the midbrain and hippocampus are found in tryptophan pyrrolase knockout mice [138]. It is also reported that some antidepressant medications boost the brain’s concentration of serotonin’s precursor, L tryptophan, by inhibition of the tryptophan pyrrolase enzyme [139].

There is an important relationship between oxidative stress and inflammation in schizophrenia and other disorders [140]. Indeed, the kynurenine pathway (Scheme 1) contains intermediate substances that are prodigious producers of oxidative free radicals [141]. Release of such radicals, by this and other means, challenges the important antioxidant role of reduced glutathione (GSH), as production of GSH may be restricted in a low-methylation environment characterized by low SAMe availability for downstream homocysteine synthesis. Since homocysteine resides at the top of the trans-sulfuration pathway to GSH synthesis, its restriction may combine with low vitamin B6 levels to restrict synthesis of GSH at the end of this pathway [142,143].

Much has been written about and reported on gastrointestinal inflammation, immune responses, altered microbiome, and the gut–brain axis in schizophrenia [144,145,146], and in this context, the role of elevated histamine deserves additional discussion. Histamine may be released from mast cells in the gut lining, causing an inflammatory reaction [147]. As outlined previously in Section 2.1 and Scheme 1, the methylation molecule SAMe plays a role in promoting histamine degradation through histamine methyl transferase (HMT) enzymes [41]. Histamine may also be degraded in the gastrointestinal lining by vitamin B6 (PLP)-dependent diamine oxidase enzyme [DAO] [148]. Therefore, in a low-methylation state, lacking SAMe availability or experiencing low vitamin B6 availability, histamine levels may be elevated [149]. Indicators of such histamine elevation have been found in the context of the *MTHFR 677 CC* wild-type genotype [81]. This genotype codes for a normally functioning MTHFR enzyme, however, CC carriers may lack adequate levels of vitamin B6 (PLP), folate, and vitamin B2 due to low dietary availability or poor vitamin absorption in the context of gastrointestinal inflammation. Moreover, riboflavin deficiency has the capacity to restrict FAD availability and, thereby, undermine production of the activated 5-MTHF form of folate produced by the FAD co-factored MTHFR enzyme. Similarly, insufficient FMN availability due to riboflavin deficiency restricts activation of vitamin B6 to PLP [67]. Since riboflavin deficiency is associated with impaired maintenance of mucous membranes and low-grade bowel inflammation [150,151], this would also contribute to the malabsorption of other nutrients such as vitamin B6 and folate. Although it is reasonable to conclude that individuals with gastrointestinal inflammation markers would experience nutrient malabsorption, direct evidence linking vitamin nutrient malabsorption with specific mental illness states, is not clearly present in the literature. This represents a research opportunity, especially since there is a reported linkage between malabsorption and gluten-induced inflammation in coeliac disease and gluten intolerance in coeliac disease and in schizophrenia [152,153,154,155].

Formaldehyde is another intermediate molecule with potential for gastrointestinal inflammation and neurotoxicity. As seen in Scheme 1, formaldehyde is the hydrogenated product of formic acid, from formate. Formate is, in turn, the product of 5,10-methylenetetrahydrofolate, which is the original product of serine, via activity of the enzyme serine hydroxy methyltransferase (SHMT). In mitochondria, formate may also be derived from dimethylglycine (DMG), which is itself a product of trimethyl glycine (TMG), which is derived from choline. Formaldehyde may be generated by gastrointestinal bacteria [156]. It induces and accelerates glycolytic flux and influences the export of the antioxidant glutathione out of astrocytes and neurons, thereby increasing their intracellular oxidative stress. However, there is little direct scientific evidence linking formaldehyde production with mental illness syndromes, and this provides another opportunity for further research [157,158].

## 4. Future Directions

Apart from addressing the problem of un-replicated studies and a broader incorporation of potential biomarkers, future research work will undoubtedly harness algorithmic and machine learning applications and adopt a systematic approach to validate and integrate genetic mechanisms with biochemical mechanisms. This will involve the investigation of multiple candidate genes alongside a broad spectrum of biochemical markers [159]. However, clinicians will need to bear in mind that genotypes do not always determine biochemical phenotypes, because, as we have seen, biochemistry contains its own inherent checks and balances. If pressed too far in one direction or another, inhibitory feedback mechanisms are applied, or enhanced activity is invoked in alternative enzyme pathways, to achieve a critical goal by other means. These biochemical adaptations and their outcomes may upset research expectations that are based on gene indicators alone. As can be seen from the compensative role of the BHMT enzyme in providing an alternative route for methionine synthesis from homocysteine, the biochemistry has back-up safety systems in place and is therefore able to maintain regeneration of methionine from homocysteine to maintain critical supply of SAMe. However, nothing in the complex matrix of interactive biochemistry is simple—and the use of BHMT in this setting unfortunately utilizes zinc, which may lead to zinc depletion, and this undermines the immune response [160,161].

Future investigations are ideally carried out in medication-naïve first-presentation participants, with further investigation of the impact of smoking and other environmental toxins [162]. Alteration of existing medication may be necessary as oral contraceptives, proton pump inhibitors, nitrous oxide, ibuprofen, and some antibiotics, as well as metformin and aspirin can inhibit methyltransferases in a manner that suppresses methylation [163,164]. Sodium valproate has also been noted to decrease folic acid levels and raise homocysteine levels [165]. Oral contraceptive use has a reported impact on vitamin B6, folate, and vitamin B12 levels [166]. The role of the thyroid hormone (Scheme 1) requires greater research attention in relation to methylation. This is because low thyroid hormone levels can restrict activity of the enzyme carrying out FMN synthesis from riboflavin [65]. This in turn restricts provision of FAD, which is required to cofactor the MTHFR enzyme and also the MAO enzyme in the first step of catecholamine metabolism. As mentioned previously, large-scale studies are also required to confirm the link between inflammatory gastrointestinal conditions and vitamin malabsorption in psychosis, as well as to investigate underlying malabsorption mechanisms relating to inflammatory molecules such as histamine, formaldehyde, and gluten.

## 5. Conclusions

This review concerns the role of the methylenetetrahydrofolate reductase (MTHFR) enzyme activity and associated biochemical pathways in schizophrenia and schizoaffective disorder. It describes *MTHFR* C677T gene - related biochemical mechanisms whereby riboflavin and its flavin derivatives interact with the methyl folate production and the ability of the MTHFR enzyme to influence vitamin activation systems and trace element levels. The potential diagnostic value of candidate markers is drawn from the broader biochemical landscape surrounding methylation mechanics. These effects combine to regulate methylation processes, modulate molecular oxidative stress defenses, influence dopamine and serotonin neurotransmitter availability, and influence gastrointestinal and brain function. From this broad landscape, potentially meaningful candidate markers and enzyme cofactors can be selected to test their capacity for predicting diagnosis and indicate treatment for schizophrenia and schizoaffective disorder. Many of these selected markers have already yielded biomarkers with high diagnostic certainty, along with indications for targeted or adjunctive treatment to reduce symptoms and prevent the relapse of persons with schizophrenia or schizoaffective conditions. Such biochemical understandings lead on to therapeutic solutions and identification of functional biochemical phenotypes and subtype classification for schizophrenia and schizoaffective psychosis. This review redefines the territory in which biochemical influences for serious mental illnesses may be found, from which biomarkers that carry implications for adjunctive treatment and management of schizophrenia by biochemical means may be selected.

## Data Availability

The data presented in this study can be made available on request from S.F.-W.
